# Novel Multiplex Bead-Based Assay for Detection of *IDH1* and *IDH2* Mutations in Myeloid Malignancies

**DOI:** 10.1371/journal.pone.0076944

**Published:** 2013-09-30

**Authors:** Velizar Shivarov, Milena Ivanova, Evgueniy Hadjiev, Elissaveta Naumova

**Affiliations:** 1 Laboratory of Hematopathology and Immunology, National Hematology Hospital, Sofia, Bulgaria; 2 Department of Clinical Immunology, Alexandrovska University Hospital, Medical University, Sofia, Bulgaria; 3 Department of Clinical Hematology, Alexandrovska University Hospital, Medical University, Sofia, Bulgaria; Queen's University Belfast, United Kingdom

## Abstract

Isocitrate dehydrogenase 1 and 2 (IDH) mutations are frequently found in various cancer types such as gliomas, chondrosarcomas and myeloid malignancies. Their molecular detection has recently gained wide recognition in the diagnosis and prognosis of these neoplasms. For that purpose various molecular approaches have been used but a universally accepted method is still lacking. In this study we aimed to develop a novel bead-based liquid assay using Locked nucleic acids (LNA)-modified oligonucleotide probes for multiplexed detection of the most frequent *IDH1* (p.R132C, p.R132G, p.R132H, p.R132L, p.R132S) and *IDH2* (p.R140Q, p.R172K) mutations. The method includes four steps: 1) PCR amplification of the targeted fragments with biotinylated primers; 2) Direct hybridization to barcoded microbeads with specific LNA-modified oligonucleotide probes; 3) Incubation with phycoerythrin coupled streptavidin; 4) Acquisition of fluorescent intensities of each set of beads on a flow platform (LuminexCorp., USA). We tested the performance of the assay on both artificial plasmid constructs and on clinical samples from 114 patients with known or suspected myeloid malignancies. The method appeared to be superior to direct sequencing having a much higher sensitivity of 2.5% mutant alleles. Applying this method to patients' samples we identified a total of 9 mutations (one *IDH1* p.R132C, seven *IDH2* p.R140Q and one *IDH2* p.R172K). In conclusion, this method could be successfully implemented in the diagnostic work-up for various tumors known to harbor *IDH1/2* mutations (e.g. myeloid malignancies, gliomas, etc.). International initiatives are needed to validate the different existing methods for detection of *IDH1/2* mutations in clinical settings.

## Introduction

In the last 5 years high-throughput and next generation sequencing allowed the in-depth elucidation of genomic complexity underlying the heterogeneous group of myeloid malignancies. This allowed the proposal of models for molecular leukemogenesis and clonal evolution but much remains to be learned about the precise mechanism of action of each mutated protein[1]. Significant progress however has been made in the elucidation of the mechanism of action of the mutations in Isocitrate dehydrogenase (*IDH*) 1 and 2 [2] genes. *IDH1* mutations affect predominantly codon R132, and *IDH2* mutations affect R140 and R172. All of them are gain-of-function mutations leading to a neomorphic enzymatic activity of the mutant peptides. It has been conclusively shown that IDH1 and IDH2 mutant enzymes catalyze predominantly the generation of an “oncometabolite” (R)-2-hydroxyglutarate (2-HG), which acts through downstream modulation of the activity of 2-HG dependent enzymes (e.g. TET family of 5-methylcytosine hydroxylases)[2]. Notably, *IDH1/2* mutations are frequently found in more aggressive myeloid malignancies (e.g. AML and MPN in transformation) and confer worse prognosis [3], [4]. Thus there is a need for *IDH*1/2 mutational status testing in the context of integrated molecular profiling [5]. For that purpose a plethora of molecular methods were adapted for the identification of *IDH1* and *IDH2* mutations in human gliomas and myeloid malignancies such as direct DNA sequencing [6], restriction fragment length polymorphism (RFLP) gel electrophoresis[7], allele specific polymerase chain reaction (AS-PCR)[8], pyrosequencing[9], real-time PCR [10], high resolution melting (HRM)[11]–[13] curve analysis, denaturing high-performance liquid chromatography (DHPLC)[14], next generation sequencing (NGS) [15] and even immunohistochemical detection of *IDH1* R132H mutant protein [8]. All the methods have certain advantages and drawbacks. Sanger sequencing is still the gold standard in the identification of mutations but in the case of somatic mutations detection its low sensitivity (∼15–20% mutant allele burden) may yield false negative results. In contrast AS-PCR and RT-PCR assays for *IDH1/2* mutations are more sensitive – around 1% mutant allele but require separate reaction for each mutation which is usually impractical or if multiplexed cannot distinguish between the specific mutation type [16]. HRM provides platform for a single tube detections of numerous mutations with a sensitivity of about 5% [11], [17].

In order to address the requirements of the clinical practice for a sensitive multiplexed and easily applicable molecular method for detection of *IDH1* and *IDH2* mutations we developed an accurate, sensitive and mid-throughput bead-based liquid assay for detection of the *IDH1* p.R132C, *IDH1* p.R132G, *IDH1* p.R132H, *IDH1* p.R132L, *IDH1* p.R132S and *IDH2* p.R140Q, *IDH2* p.R172K mutations. We validated our assay on clinical samples from patients with myeloid malignancies.

## Materials and Methods

### Ethics Statement

The study was conducted in accordance with the principles of the Declaration of Helsinki. Written informed consent was obtained from all patients. No specific approval for the study was required as all blood and bone marrow sampling as well as molecular testing was part of the routine diagnostic procedures already approved by the Institutional Review Board (IRB) at Alexandrovska University Hospital, Sofia, Bulgaria.

### Patient samples

A total of 114 peripheral blood or bone marrow samples of consecutive patients with known or suspected myeloid malignancies were collected between January 2010 and December 2012. Patients were classified according to the WHO criteria as follows: Acute myeloid leukemia (AML) (n = 21), Chronic myeloid leukemia (CML) (n = 1), Myelodysplastic syndrome (MDS) (n = 6), Myeloproliferative neoplasm (MPN) (n = 75), Overlap MPN/MDS (n = 5), and others with suspected but unproven MPN (n = 6).

### DNA extraction

All samples were collected using sodium citrate-containing blood sampling tubes (BD Biosciences) and stored at room temperature for no more than 4 hours before processing. Genomic DNA was extracted from whole blood using a iPrep (Invitrogen) automated system. Genomic DNA samples were stored at −20°C before further analyses.

### *IDH1* and *IDH2* exon 4 sequencing

Fragments of *IDH*1 and *IDH2* exon 4 were amplified using the following primers: IDH1 (forward): 5′- GGATGCTGCAGAAGCTATAA and IDH1 (reverse): 5′-TTCATACCTTGCTTAATGGGTGT, IDH2 (forward) 5′-AATTTTAGGACCCCCGTCTG, IDH2 (reverse): 5′- GGGGTGAAGACCATTTTGAA. Amplification was performed using: 100 ng genomic DNA; 1.5 U Taq polymerase (Invitrogen); 3 mM MgCl2; 0.2 mM dNTP mixture; and 10 pmol of each primer in 25 µl reaction. The amplification conditions were as follows: 95°C for 5 min; 35 cycles −95°C for 30 sec, 58°C for 40 sec, 72°C for 2 min and final extension at 72°C for 10 min. PCR products thus generated were purified by Exo-SAP (Applied Biosystems, USA) and sequenced directly. The sequencing reaction was conducted in a 10-µL final volume using 2 µL of the purified PCR product, 3.2 pmol of one the PCR primers and 2 µL of Big Dye terminator cycle-sequencing kit v3.1 (Applied Biosystems, USA). The sequencing program was a 25-cycle PCR program (denaturation at 96°C for 10 s; annealing at 50°C for 5 s and elongation at 60°C for 4 min). The sequence detection was conducted using the ABI Prism 3100 Genetic Analyzer (Applied Biosystems, USA).

### Bead-based assay

The fragments encompassing the *IDH1 and IDH2* exon 4 were amplified from either genomic or plasmid DNA samples using 5′-botinylated forward primer. The same primers and PCR conditions as described above for the sequencing analysis were applied. Genotyping was performed by direct hybridization with 6 and 4 LNA-modified oligonucleotide probes, specific for the wild type the mutant alleles of *IDH1* and *IDH2* genes, respectively (Exiqon, Denmark): The sequences were *IDH1* (p.R132: 5′-AAGCATGACGACCTATG; p.R132C: 5′-AAGCATGACAACCTATG; p.R132S: 5′-AAGCATGACTACCTATG; p.R132G: 5′-AAGCATGACCACCTATG; p.R132H: 5′-AGCATGATGACCTATG; p.R132L: 5′-AGCATGAAGACCTATG) and IDH2 (p.R140: 5′-GATGTTCCGGATAGTTC; p.R140Q: 5′-GATGTTCTGGATAGTTC; p.R172: 5′-CGTGCCTGCCAATGGT; p.R172K: 5′-CGTGCTTGCCAATGGT). All probes were synthesized with 5′-amino group and 20 bases oligonucleotide spacers to allow covalent binding to carboxylated microbeads (Luminex, USA). Immobilization of the amine modified probe to carboxylated surface of the beads was completed using a standard carbodiimine-coupling procedure. Mixtures of 5 sets of coupled microspheres were prepared by combining equal volumes of each set of beads. Approximately, 160 microspheres of each set/µl were used for analysis of one DNA sample. Five µl of PCR product; 20 µl Hybridization buffer (Wakunaga Pharmaceuticals); 3 µl Bead mixture and 2 µl SAPE (Wakunaga Pharmaceuticals, Hiroshima, Japan) were combined in a well of a Thermowell 96-well plate and hybridization was performed at 66°C, 68°C or 70°C for 30 min in a thermocycler (Applied Biosystems, USA). After incubation, 75 µl Washing buffer (Wakunaga Pharmaceuticals, Japan) were added to each reaction and the plate was centrifuged at 4000 rpm for 2 min. The supernatant was removed and the microspheres were resuspended in 75 µl Washing buffer (Wakunaga Pharmaceuticals, Japan). The samples were analyzed on a LabScan200 machine (Luminex, USA). A minimum of 100 events per bead region of interest were collected. For each set of reactions a background control of a sample containing only the respective sets of beads was included. The background mean flourescence intesity (MFI) values were subtracted from the MFI values for each sample. The resulting values were used for the calculation of the relative fluorescence indices for each genomic location as follows: Index (mutant allele*_i_*)  = [MFI (mutant allele*_i_*)/[MFI (mutant allele_1_) + … +MFI (mutant allele*_n_*) +MFI (wild type allele)], where n equals 5 or 2 for *IDH1* p.R132 and *IDH2* p.R140 and p.R172, respectively.

### Determination of the cut-off index values of the assay

Sensitivity and specificity of the assay for each mutant as well as the optimal threshold values for mutant allele indices were determined using Receiver Operating Characteristic (ROC) analysis[18]. All indices for positive and negative samples for each mutant from five independent runs were used for the analysis. Analysis of the sensitivity in terms of percent mutant allele burden was performed by plotting the range of indices for each mutant at concentration 1%, 2.5% and 5% versus the already determined cut-off value. ROC analysis and plotting were performed using the “ROCR” package[19] for R statistical environment.

## Results

### Determination of the hybridization conditions

**Figure 1:**
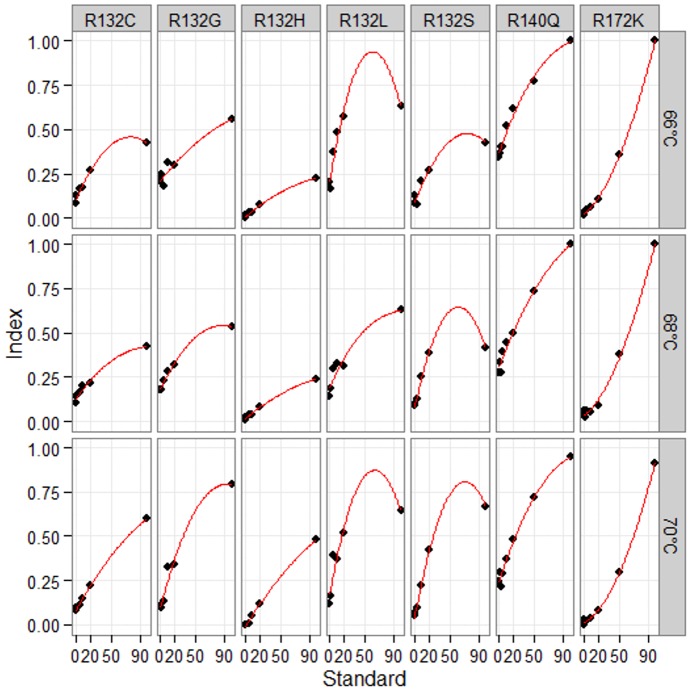
Standard curves for each mutant at the three different hybridization temperatures.

In order to determine the optimal hybridization conditions we prepared dilutions of the 100% mutant plasmid with 100% wild type plasmid to generate reference samples with each allele at levels of 100, 50, 20, 10, 5, 2.5, 1 and 0%. As the Tm for all the probes was estimated to around 72°C hybridization reactions for all the standards were set up at 66°C, 68°C and 70°C. Mutant allele index for each sample was calculated and standard curves were generated for each mutant at every hybridization temperature (Fig. 1.). Several models were tested for best curve fitting – linear regression, polynomial (quadratic) regression and hyperbolic regression. Comparison between the three models is shown at Table S1 and the curves for hyperbolic regression model are shown at Fig. S1. Quadratic regression was chosen because it showed the highest R^2^ values. Furthermore, coefficient of determination (R^2^) for each quadratic curve was calculated showing that the highest R^2^ values were obtained for the 70°C level but the difference was not statistically significant (p = 0.507) (Fig. S2B). As high hybridization temperature can increase specificity while reducing the intensity of the fluorescent signal we calculated the delta MFI value for each allele versus every other (Fig. S2A). Expectedly, the highest delta values were observed for the lowest temperature tested (i.e. 66°C) and lowest for the highest temperature (i.e. 70°C), (p<0.0001). As a compromise between R^2^ values and delta values we selected the 68°C for hybridization temperature for all subsequent runs of the assay.

### Determination of the index threshold value and assay sensitivity

**Figure 2:**
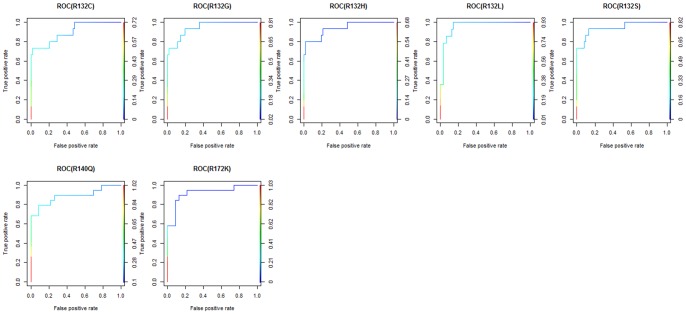
ROC curves for all mutants. TPR-true positive rate; FPR-false positive rate.

**Figure 3:**
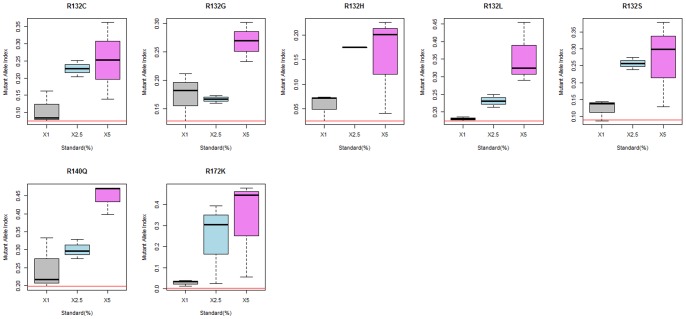
Graphical demonstration of the sensitivity of the assay for each mutant as expressed by percent mutant allele burden on wild type background. Data for the boxplots are from five independent experiments. The red lines denote the index cut-off values determined in the ROC analysis as described in the text.

To determine the mutant allele index threshold values we used Receiver Operating Characteristic (ROC) analysis. ROC curves for all mutants are shown at Fig. 2. The determined areas under the curve (AUC) were over 0.9 for the following mutants: p.R132C (0.902), p.R132G (0.945), p. R132H (0.939), p. R132L (0.963), p. R132S (0.944), and p.R172(0.92). Only one mutant p.R140Q had AUC for the ROC curve under 0.9 (0.888). As the positive results from this assay would be expected to diagnose oncological disease the cut-off index value for every mutant was determined as the value providing the maximum specificity at 100% sensitivity (Table 1). To determine the sensitivity of the assay for each mutant as expressed in percent mutant allele burden we assayed plasmid samples with wild type only and 1%, 2.5% and 5% of each mutant plasmid on wild type background. Sensitivity was defined as the lowest mutant allele burden whose range of index values was above the determined respective cut-off index. As shown in Fig. 3 the sensitivity was 2.5% for all *IDH1* mutants and for *IDH2* p.R140Q and 1% for *IDH2* p.R172K mutant.

**Table 1:** Cut-off values determined based on the ROC analysis. TPR-true positive rate (equivalent to sensitivity), FPR-false positive rate (equivalent to 1-specificity).

Mutant	TPR	FPR	Cut-off
R132C	1	0.483871	0.075788
R132G	1	0.354839	0.128681
R132H	1	0.492063	0.026123
R132L	1	0.142857	0.174801
R132S	1	0.532258	0.088561
R140Q	1	0.782609	0.198606
R172K	1	0.73913	0.003246

### Concordance of the bead-based assay with direct sequencing using genomic DNA

**Figure 4:**
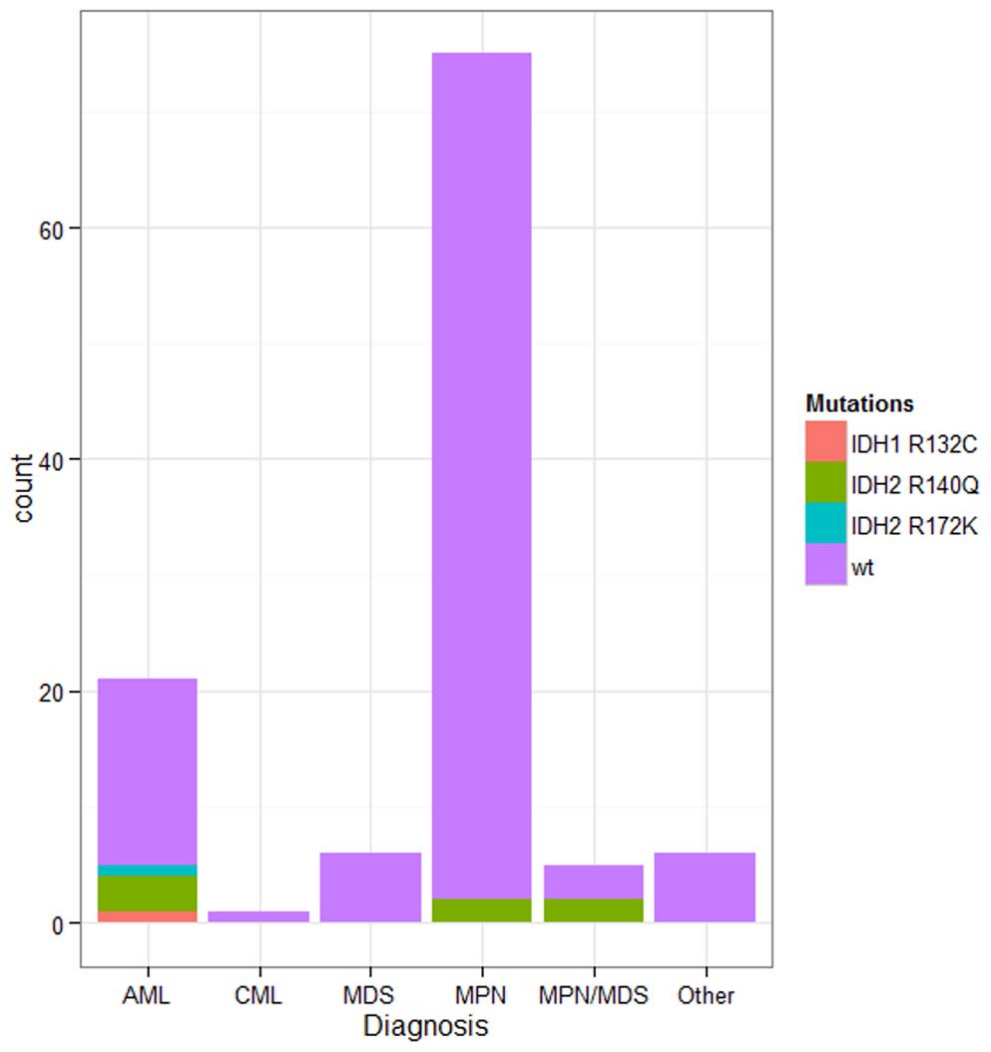
Distribution of mutated cases per disease entity.

**Figure 5:**
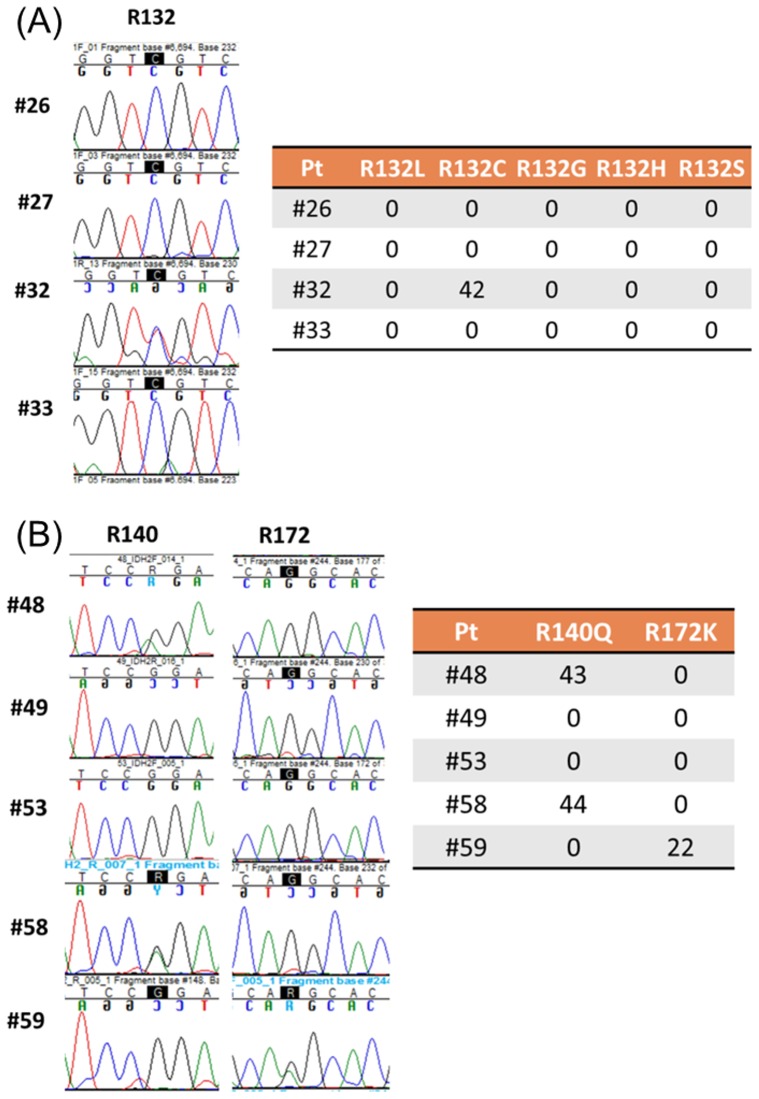
Comparison of the read-out of the bead-based assay and the Sanger sequencing in selected patients' samples for *IDH1* (A) and *IDH2* (B) genes.

We further tested the performance of our bead-based assay on a set of genomic DNA samples from 114 patients with known or suspected myeloid malignancies. We identified a total of 9 mutations (one *IDH1* p.R132C, seven *IDH2* p.R140Q and one *IDH2* p.R172K). The distribution of mutant cases per disease entity is shown in Fig. 4B. In accord with previous reports the frequency of IDH1/2 mutations was highest in AML cases (23.8%)[20]. Indeed, we confirmed the presence of the mutation by direct sequencing (Fig. 5) Notably, the results from the bead-based assay were completely concordant with the Sanger sequencing. In addition, we sequenced all remaining cases determined as *IDH1/2* mutation negative by the bead-based assay. We did not identify any case positive for *IDH1/2* mutation on direct sequencing. This further validated our assay in clinical settings.

## Discussion

The bead-based flow platform (LumiexCorp., USA) has recently been utilized for a number of biomedical applications [21]. It has also been demonstrated as applicable for detection of oncogenic somatic mutations such as *KRAS* and *BRAF* [22], [23], *JAK2* and *MPL* [24]–[27], *NPM1* [28] and for detection of chromosomal translocations [29], [30]. All these assays were based on a straightforward PCR amplification of the target region and subsequent direct hybridization with specific oligonucleotide probes. The great advantages of the system are the possibility for multiplexing, mid-throughput samples processing and labour and time saving features [21]. Though it is sometimes considered a semiquantitative technique we have recently demonstrated that this platform can quantify mutant allele burdens of mutations associated with MPN [24]–[26]. This feature may not be of clinical advantage in every instance as it is usually sufficient to demonstrate the presence/absence of a somatic mutation to diagnose cancer. Of particular practical importance however remains the sensitivity of the assay as usually a tumor sample represents a mixture of normal and malignant cells and the latter might be quite rare on some occasions.

Here we adapted the Luminex flow platform for the detection of the recently identified mutations in exon 4 of *IDH1* and *IDH2* genes. These mutations are frequently found in gliomas, acute myeloid leukemias, chondrosarcomas and cholangiocarcinomas. This makes their testing of particular clinical importance for diagnostic purposes. Furthermore, some reports demonstrated the prognostic importance of IDH mutations in myeloid malignancies such as AML [4] and MPN [3]. We validated our multiplex assay for detection of *IDH1* and *IDH2* mutations on plasmid vectors harboring wild type or mutant alleles. As expected the assay appeared to be quantitative with clinically relevant sensitivity of 2.5–5.0% which is superior to direct sequencing and comparable to HRM methods. Further enhancement of the sensitivity of our assay is possible through coupling with initial COLD-PCR amplification as already reported for *IDH1* mutations detection [31], [32]. This would be of value if *IDH1/2* mutational status is to be tested for detection of minimal residual disease [33]. Besides, our method is applicable not only to patients with myeloid malignancies but also to those with glial tumors or chondrosarcomas or cholangiocarcinomas as in its current format it covers all most frequent mutations.

## Conclusions

In conclusion, our bead-based assay for detection of *IDH1* and *IDH2* exon 4 is a fast method taking up to 5 hours from DNA extraction to data acquisition. Furthermore, it is quantitative and robust and yields a sensitivity of 2.5% mutant allele. It allows also a mid-throughput analysis in 96 or 384 well plates. This method can be easily implemented in the algorithmic approaches for integrated mutational profiling of myeloid malignancies either alone or after further extend the multiplexing for single-tube detection of various other mutations of diagnostic and prognostic importance. Further optimization of our assay and independent verification of its quantitative power at low and very low allele burden levels would obviously require direct comparison with other molecular techniques such as high-resolution melting (HRM) analysis and Next Generation Sequencing (NGS). Finally, international initiatives would be of particular importance to validate in a comparative fashion the different existing methods for detection of *IDH1/2* mutations in clinical settings [34].

## Supporting Information

Figure S1 **Curve fitting with hyperbolic regression model for all mutants obtained at 68°C hybridization.**
(PDF)

Figure S2 **Determination of the optimal hybridization temperature.** (A) Distribution of the deltaMFI values for all mutants at the three different temperatures (p<0.0001 on Kruskal-Wallis test). (B) Distribution of the R^2^ values for all mutants at the three different temperatures (p = 0.507 on Kruskal-Wallis test).
(PDF)

Table S1 **Comparison of the performance parameters of three curve fitting models for all mutants at the 68°C hybridization temperature.**
(PDF)
