# Supplementary figures and images for: Novel Multiplex Bead-Based Assay for Detection of IDH1 and IDH2 Mutations in Myeloid Malignancies
Source: PLoS One. 2013 Sep 30;8(9):e76944. doi: 10.1371/journal.pone.0076944 (PMC3786925; doi:10.1371/journal.pone.0076944)

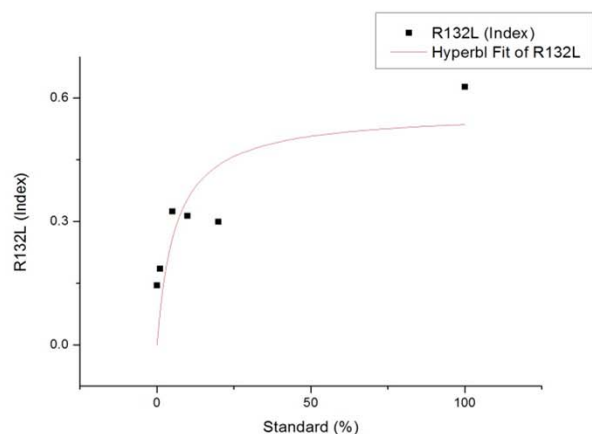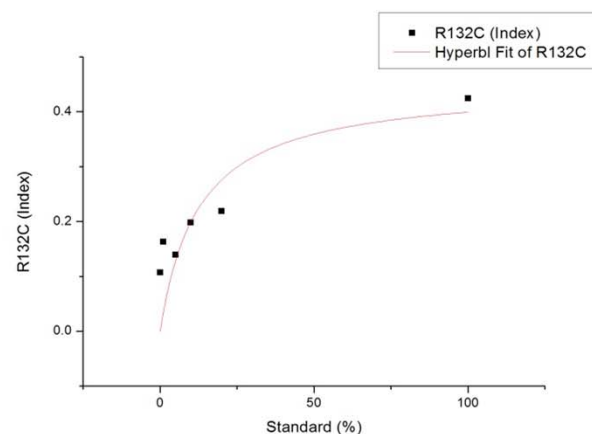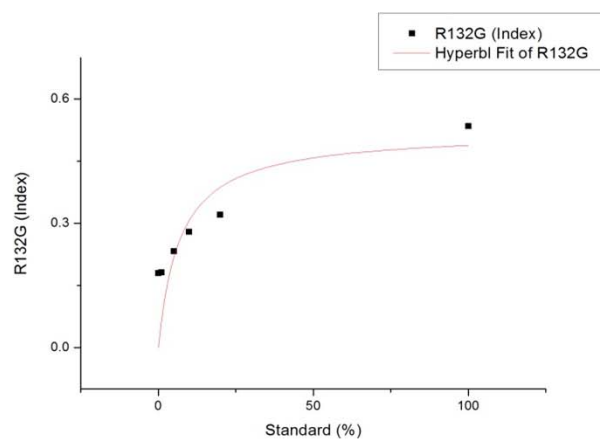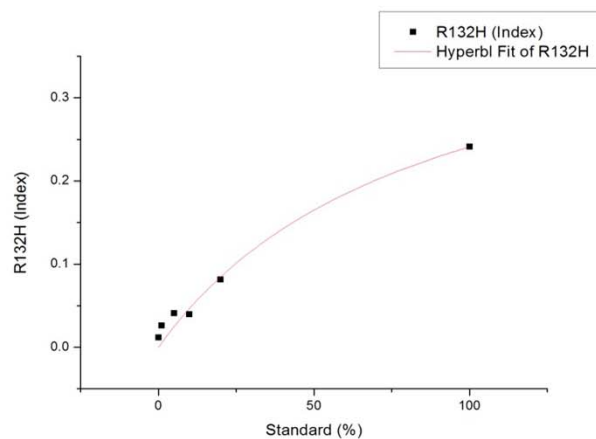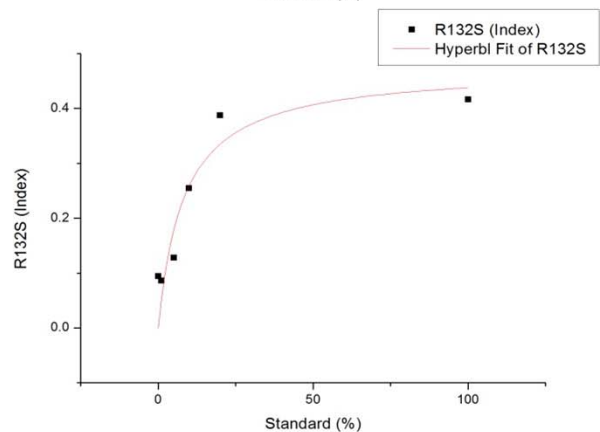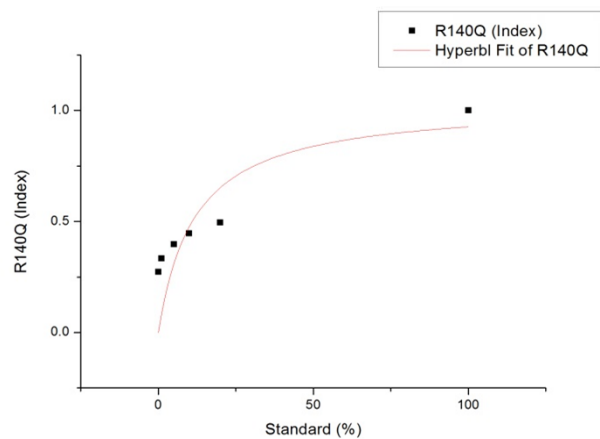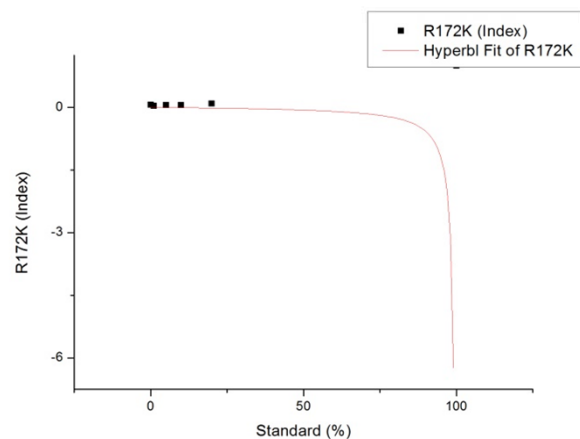

Supplement: Figure S1 — Curve fitting with hyperbolic regression model for all mutants obtained at 68°C hybridization. (PDF) [file pone.0076944.s001.pdf]

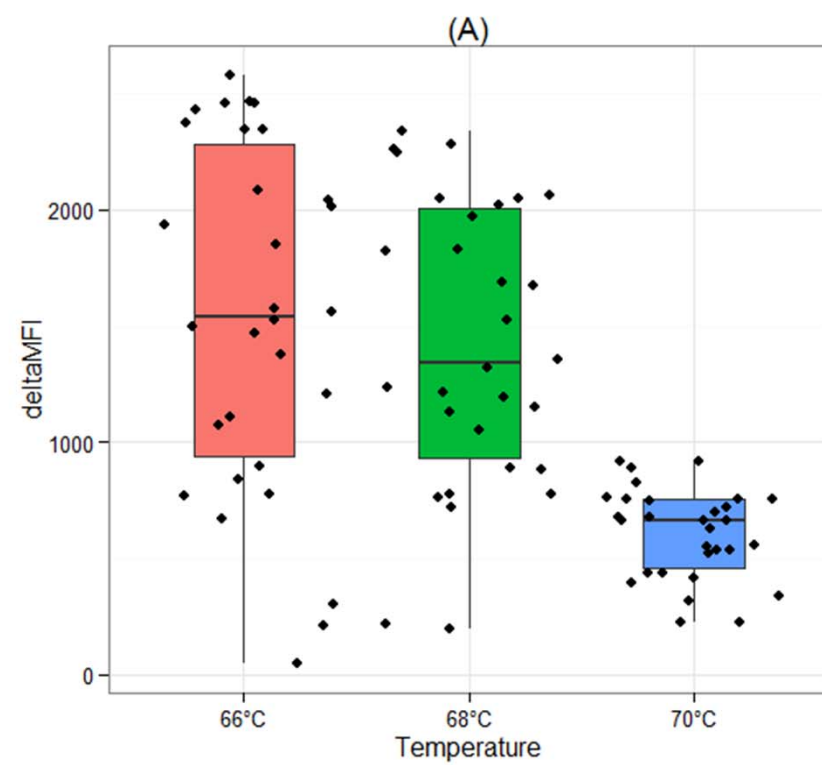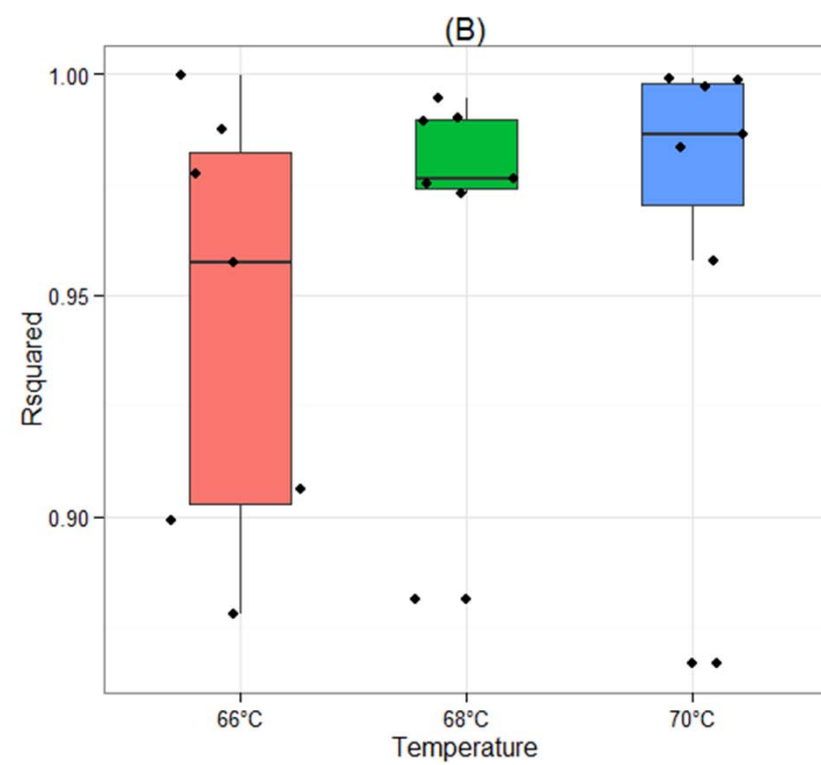

Supplement: Figure S2 — Determination of the optimal hybridization temperature. (A) Distribution of the deltaMFI values for all mutants at the three different temperatures (p<0.0001 on Kruskal-Wallis test). (B) Distribution of the R2 values for all mutants at the three different temperatures (p = 0.507 on Kruskal-Wallis test). (PDF) [file pone.0076944.s002.pdf]
