# Supplementary material for: Novel Multiplex Bead-Based Assay for Detection of IDH1 and IDH2 Mutations in Myeloid Malignancies
Source: PLoS One. 2013 Sep 30;8(9):e76944. doi: 10.1371/journal.pone.0076944 (PMC3786925; doi:10.1371/journal.pone.0076944)
Supplement: Table S1 — Comparison of the performance parameters of three curve fitting models for all mutants at the 68°C hybridization temperature. (PDF) [file pone.0076944.s003.pdf]

Model	Linear regression		Polynomial (quadratic) regression		Hyperbolic regression	
Parameter	Mean (squared residuals)	R^2	Mean (squared residuals)	R^2	Mean (squared residuals)	R^2
R132C	0.06103	0.94202	0.03105	0.95031	0.14638	0.36864
R132G	0.08305	0.92593	0.04385	0.98912	0.26697	0.24251
R132H	0.03619	0.98594	0.01819	0.99008	0.03404	0.96916
R132L	0.12674	0.85322	0.06468	0.83458	0.33862	0.43243
R132S	0.06298	0.46810	0.05392	0.97284	0.20267	0.81832
R140Q	0.33455	0.97313	0.16908	0.98173	0.80660	0.35353
R172K	0.71986	0.97070	0.36831	0.99882	0.49775	-0.33333
